# Highlights from *e*cancer Choosing Wisely Nepal 2022: critical appraisal skills for evidence-based practice, 24th–25th September 2022, Kathmandu, Nepal

**DOI:** 10.3332/ecancer.2022.1478

**Published:** 2022-11-28

**Authors:** Bishesh Sharma Poudyal, Soniya Dulal, Ramila Shilpakar, Bishal Gyawali

**Affiliations:** 1 Clinical Hematology and Bone Marrow Transplant Unit, Department of Medicine, Civil Service Hospital, Kathmandu 44600, Nepal; 2 Department of Internal Medicine, B.P. Koirala Institute of Health Sciences (BPKIHS), Dharan 56700, Nepal; 3 Department of Clinical Oncology, National Academy of Medical Sciences, Bir Hospital, Kathmandu 44600, Nepal; 4 Queen’s Global Oncology Program, Department of Oncology, Queen’s University, Kingston, ON K7L 3N6, Canada; 5 Division of Cancer Care and Epidemiology, Queen’s Cancer Research Institute, 10 Stuart Street, Level 2, Queen’s University, Kingston, ON K7L 3N6, Canada

**Keywords:** critical appraisal, evidence-based medicine, systematic reviews, guidelines, peer review, mentorship, global oncology

## Abstract

The *e*cancer Kathmandu 2022 workshop on the 24th–25th September 2022 was the first *e*cancer conference organised in Nepal, a Southeast Asian nation sandwiched between India and China. It was focused on critical appraisal skills for evidence-based practice and was organised in partnership with the Karnali Academy of Health Sciences and the Civil Service Hospital from Nepal, and the Queen’s Global Oncology Program from Canada. The workshop emphasised the need for critical thinking in understanding clinical research, and also motivated the delegates to undertake meaningful clinical research relevant to the local setting. The sessions highlighted the features of a good clinical research, identify pitfalls in the reporting of clinical trials, implementation of the research into locally relevant practice and development of local clinical guidelines. Furthermore, the faculty also discussed how to write a good scientific paper, the do’s and don’ts of a systematic review and meta-analysis, the role of peer-review and how to do one properly and what do editors look for in evaluating papers submitted for publication. The audience learned the importance of finding a good mentor and fostering local and international collaboration. The local faculty also highlighted their own personal journeys and how mentorship and global collaboration played an important role in their own academic career. The enthusiastic panel discussion was a highlight of the programme where the delegates learned about several important topics from the faculties, such as work–life balance, the role of mentorship in building careers and building networks.

## Introduction

Nepal is a small low-middle income country in Southeast Asia undergoing an epidemiological transition from communicable to non-communicable diseases in recent years. To address this challenge, several subspeciality training programmes in several disciplines such as oncology, haematology, cardiology, etc., are now established in the country [1]. Through the impact of social media, globalisation and international collaboration, several cutting-edge interventions and therapeutics are also now available for treatment of patients in Nepal. However, despite all this progress, there are negligible opportunities for physicians to get trained on evidence-based practice and critical appraisal skills that are fundamental to all these disciplines. Critical appraisal skills are not formally taught during medical school, residency or fellowship training. To fill this gap, a workshop on ‘Critical Appraisal Skills for Evidence-Based Practice’ was organised by the *e*cancer Global Foundation in collaboration with the Karnali Academy of Health Sciences, Nepal; Queen’s Global Oncology Program, Canada and the Civil Service Hospital, Nepal as a 2-day event on the 24th and 25th of September at Gokarna Forest Resort, Kathmandu, Nepal under the co-presidency of Dr Bishesh Sharma Poudyal and Dr Bishal Gyawali. One hundred and twenty-four young physicians across different medical specialities as well as medical students, medical school graduates, residents, pharmacists, public health students and nursing staff enthusiastically participated in the meeting in addition to 11 speakers from different countries with different specialists. The aim of the meeting was to promote critical thinking for evidence-based medicine and to encourage healthcare professionals to doing medical research.

## Highlights of Day 1

Day 1 of the event opened with an inauguration ceremony accompanied with traditional Nepali music. Dr Bishesh Poudyal, Clinical Haematologist and bone marrow transplant physician from the Civil Service Hospital, Kathmandu, Nepal, then welcomed all the faculties and delegates. Dr Bishal Gyawali, Queen’s Global Oncology Program, Kingston, Canada, then gave an introduction to the event and discussed why such an event was organised. He also announced an award in honour of his parents to be given annually to medical trainees of Nepal who have published excellent medical research despite the constraints in the country.

Dr Ian Tannock, from Princess Margaret Cancer Centre, University of Toronto, Canada, started the education session with a talk on clinical research. He discussed what is clinical research, how to think about clinical research and how to conduct clinical research. He tried to encourage young health professionals in Nepal to develop research ideas and conduct research that is relevant to the local context. He emphasised the importance of testing low-cost strategies for existing therapies, such as using a lower dose or a less-frequent schedule of therapy such as targeted or immunotherapy drugs in cancer care [2, 3]. He emphasised that opportunities for simple but meaningful research existed in resource-constrained settings, and that clinical research did not always need to be big and fancy [4].

Dr Bhawna Sirohi, from Balco Medical Centre, Raipur, Chhattisgarh, India, talked about strategies for conducting research in low- and middle-income countries (LMICs) [5]. She suggested that, to do research, one had to identify what was important in the local setting. She highlighted the importance of cancer registry to know the burden of disease in the country. Next step is to build the research agenda, focusing on screening, prevention and development of resource-stratified protocol that is relevant to that country. Then, the last step is to establish research capacity. She emphasised to direct one’s efforts towards epidemiology and outcome-based research, that is applicable to that region.

Dr Soniya Dulal, Medical Oncologist from B.P. Koirala Institute of Health Sciences (BPKIHS), Dharan, Nepal, talked about the priorities of cancer research in Nepal. She mentioned the need for good-quality data on the incidence, prevalence, morbidity and mortality of common cancers in the community because the data from the National Cancer Registry was underrepresentative. She emphasised that despite challenges in terms of research capacity, we could address them by generating our own local data relevant to our population via collaborative research network [6]. She highlighted that, our priorities in research should focus on epidemiology, screening, prevention, palliative care, developing resource-stratified guidelines, health policy and implementation. Lastly, she gave an example of how global collaboration helped her establish the Medical Oncology unit in BPKIHS, Dharan to develop their own region-specific data despite limited resources and challenges [7, 8].

Dr Bishesh Sharma Poudyal talked about his experience of doing research in Nepal. He started his talk by stating a strong need to conduct clinical research to identify and implement effective and cost-effective treatment plans. He added that because of a difference in disease pattern and priority, clinical research conducted in high-income countries did not represent the need for patients from low-income countries. He further showed data that only 21 of 1,556 new drugs marketed between 1975 and 2004 were targeting diseases specifically prevalent in poor and low-income countries and therefore emphasised the need for conducting clinical research to address the local disease burden in LMICs [9]. He also mentioned that researchers from LMIC’s should take it as an opportunity and should collaborate with each other to generate good evidence [10]. He further discussed the challenges of conducting research in a LMIC setting. He mentioned that there is no luxury of protected time for conducting research in LMICs primarily because of the high patient burden and that most of the research-related work needed to be done after standard office hours. He also stressed that at times LMICs needed clinical research to validate the findings from high-income countries. He gave an example of his aplastic anaemia paper which showed a staggering low response rate of antithymocyte globulin and cyclosporine in the paediatric subgroup compared to high-income countries and how this paper has changed the treatment guidelines for aplastic anaemia in his institution [11]. He also stressed the importance of publishing the manuscript in free online journals and explained how it could help patients to exercise their rights to decide on their treatment plans.

Namita Ghimire, from the Nepal Health Research Council (NHRC) gave a brief overview of the role of the Nepal health research council in facilitating medical research in Nepal. She explained the roles and responsibilities of the NHRC. She described the steps of ethical review process in detail. She highlighted the fact that the NHRC is the national regulatory body to regulate all health research in Nepal and all researchers should follow the National Ethical Guidelines for Health Research in Nepal.

Dr Christopher Booth, from Queen’s University, Kingston, Canada, talked about the role of surrogate endpoints in clinical trials. He emphasised that only two end points really mattered to patients – increase in overall survival and improvement in quality of life. He stressed the fact that progression free survival was not a valid surrogate for survival [12] or quality of life [13]. He also pointed out publication bias in leading oncology journals against authors from LMICs [14]. He showed evidence that cancers of ‘poverty’, i.e., those cancers occurring in LMICs, are underrepresented in clinical trials globally (e.g. cervix, head & neck cancer) [15]. He highlighted that there was a real crisis of ‘value’ in cancer care: high drug prices, low value interventions and questionable endpoints are responsible for this crisis. He then shared insights on how to write a good academic paper to get it published in good journals. He shared his own experience on how journal rejection, which is a part of the process, helps make your research better. He shared practical tips on working with co-authors and responding to peer reviewers’ comments.

Dr Bishal Gyawali, from Queen’s University, Kingston, Canada, talked about pitfalls in the interpretation of clinical trials and shared tips on how to read clinical trial publications [16]. He discussed that abstracts did not always give the full picture, and the need to study and understand methods section clearly [17]. He also discussed the pervasive conflicts of interest in drug trials and how that can lead to bias in reporting, and as a result, the need for us to study trial publications with a critical eye [18]. Methodologically, he encouraged the participants to think about the appropriateness of the control arm, endpoints used and issues related to crossover design, post-protocol therapies and subgroup analyses [19]. He then highlighted the need not to be misled by terms that downplay toxicities such as ‘tolerable side effects’ [20]. He encouraged the audience to critically evaluate all trials relevant to their practice, even if they were published in reputed high-impact journals.

Day 1 ended with an extensive Panel discussion, moderated by Dr Bishal Gyawali and Dr Soniya Dulal. The session was very interactive and had enthusiastic participation from the attendees. The participants shared their challenges in conducting research and the faculties gave their opinion and guidance on addressing those challenges.

In a parallel session on Day 1, Dr Mahima Pant, Radiation Oncology resident from the National Academy of Medical Sciences (NAMS), Bir Hospital, Nepal, talked about Intraoperative Radiotherapy (IORT) and highlighted that intraoperative radiation therapy is an emerging treatment option in the field of oncology as it helps to deliver high dose of radiation to target areas and minimise dose to surrounding normal areas, which is a major aim of targeted radiation therapy. Adjuvant radiation therapy is usually given after adjuvant chemotherapy so can be delayed for months, and thus IORT omits this. It has shown promising results in treatment of early breast cancers and irradiation in Head and Neck cancer; however, more phase III data are awaited to establish its role in various cancers.

## Highlights of Day 2

After the very insightful and interactive sessions on Day 1, day 2 of Critical Appraisal Skills For Evidence-Based Practice event started with a welcome remarks by Dr Gyawali. Dr Ian Tannock began day 2 with a discussion on how to read a clinical trial critically. He explained in detail about the different aspects of responsible research publication that the readers should be aware of [21]. He especially emphasised the importance of analysing whether the results or benefits are overemphasised and toxicities are underemphasised [22]. Delivering the best possible healthcare requires a reliable evidence-base of research publications. So, it is very important that before we take a result from publication to clinical practice, we analyse if the methods are clear and unambiguous and the results are presented honestly without fabrication, falsification or inappropriate data manipulation [23].

Dr Bishal Gyawali then talked on how to read (and do) a systematic review and a metaanalysis. He discussed several Do’s and Don’ts of a systematic review and meta-analysis and clarified several misconceptions [24]. Systematic reviews and meta-analysis are great opportunities to answer important questions with limited resources, and thus ideal for trainees in Nepal as a start to get exposure to medical research and publication. However, the most important parameter was the importance of the research question, a systematic search strategy and the decision on whether or not to pool the overall results.

Dr Scott Berry from Queen’s University, Kingston, Canada, talked about developing and reading clinical guidelines. Dr Berry initially highlighted the importance of guidelines especially in cancer care. He then discussed evidence synthesis from trials and the importance of using this data in a clinical context to develop the guidelines. He stressed the importance of making high-quality comprehensive evidenced based practical guidelines based on a rigorous review of evidence and assessment of the quality of those evidences. He gave example of how clinical guidelines were developed in Ontario which is an extensive process requiring expertise and time [25]. He focused on the importance of adapting the guidelines from the international arena and then developing guidelines in the local context keeping in mind the effectiveness, cost, availability and value of different treatment options. Dr Berry also highlighted the impacts of locally adapted guidelines by discussing the model being used by the National Cancer Grid of India and how that could be an example for other LMICs like Nepal [26].

Dr Ophira Ginsburg from National Cancer Institute – Center for Global Health, USA, talked about how to do a good peer-review and the steps leading to it. She discussed about what a good peer review is, why it is important and how we should be actually doing it. She also emphasised the advantages of doing a peer review and how it can help us advance our career as researchers. Dr Ginsburg explained in detail about the various steps of peer review process. She gave insights into the science and art of the peer review process. She also talked in brief about the predatory journals we should be careful about to avoid a waste of our time and energy. She pointed out that a good peer review is very essential for differentiation of good science which once published affects not only the research community but also, indirectly, society at large. The value and benefits of research are vitally dependent on the integrity of research and publication is the final stage of research, so the peer review process is an important responsibility for ensuring the integrity of the research literature.

Professor Richard Sullivan, from Kings’ College, London, UK, discussed another important aspect of literature review from the editors’ lens. Journal editors have responsibilities for ensuring the integrity of the research literature, so Professor Sullivan talked about editors’ responsibilities on publishing research. He explained in detail about editors’ responsibilities towards authors as well as reviewers and readers. He discussed publication processes, authorship disputes and conflict of interests in detail.

Dr Ramila Shilpakar from the NAMS, Bir Hospital, Nepal, shared her personal experience of various benefits of international collaboration in cancer care. She emphasised that just like cancer which respects no boundaries, cancer care should be available equally and affordably irrespective of where we live. She further added that international collaboration and partnership are of paramount importance to achieve this goal of the cancer ground shot [27]. She stressed that everyone working in the field of oncology should work on improving patient care not only locally but globally as well and working together is the key to achieving control over this global pandemic of cancer [28]. She talked about her collaboration experience and explained it has been rewarding in gaining knowledge and how it has helped her to improve care in the entire spectrum of cancer care including prevention, early detection, treatment and palliative care [29]. She reiterated that conquering cancer takes a collective effort [6].

Dr Bishesh Sharma Poudyal talked about the value of mentorship and friendship in developing an academic career. He walked us through his inspiring and unique experience of international mentorship in improving his clinical practice and expanding the horizons of patient care [30].

Dr Soumitra Datta from Tata Medical Center, Kolkata, India, talked about managing work–life balance as an academic physician. He drew attention to the very important but overlooked aspects of physician life. He highlighted the importance of creating a balance between clinical load and research. Dr Datta shared various tips of avoiding burnout to the audiences especially the young aspiring researchers about seeking a balance between huge patient loads and research career. He emphasised the importance of achieving a balance in career as well as family life for a happy life. It was an engaging discussion on understanding and realising our priorities in our careers and identifying when we need help for burnout. He also discussed various ways we can help ourselves avoid burnout.

There was again a very enthusiastic interactive panel discussion at the end of Day 2 to allow the participants to speak their mind and ask questions, moderated by Dr Gyawali. Most of these discussions centred around finding a good mentor and the qualities of a good mentor.

Dr Ramesh Bhattarai, the Dean of Karnali Academy of Health Sciences, Nepal, concluded the event by delivering his concluding remarks highlighting the need to run such programmes regularly, thanking the organisers for hosting a meaningful event, and the participants for their enthusiastic participation.

## Conclusion

The *e*cancer Critical Appraisal event was well attended by health professionals (medical students, medical school graduates, residents, pharmacists, public health students, nursing staff as well as doctors from various disciplines). The overarching theme that came from this meeting was the need to identify local research priorities that could be accomplished in the local setting. The value of mentorship and collaboration in developing an academic career in a LMIC was highlighted. Identifying good research from bad research was discussed and tips on conducting systematic reviews, peer-review and editorial processes were shared. Persistence, motivation and working together are key to having more high-quality impactful research from LMICs.

## Conflicts of interest

None.

## Funding

None.
